# Use of all cause mortality to quantify the consequences of covid-19 in Nembro, Lombardy: descriptive study

**DOI:** 10.1136/bmj.m1835

**Published:** 2020-05-14

**Authors:** Marco Piccininni, Jessica L Rohmann, Luca Foresti, Caterina Lurani, Tobias Kurth

**Affiliations:** 1 Institute of Public Health, Charité - Universitätsmedizin Berlin, 10117 Berlin, Germany; 2 Centro Medico Santagostino, 20127 Milan, Italy

## Abstract

**Objective:**

To quantify the impact of coronavirus disease 2019 (covid-19) on all cause mortality in Nembro, an Italian city severely affected by the covid-19 pandemic.

**Design:**

Descriptive study.

**Setting:**

Nembro, in the Bergamo province of Lombardy, northern Italy.

**Population:**

Residents of Nembro.

**Main outcome measures:**

Monthly all cause mortality between January 2012 and April 2020 (data to 11 April), number of confirmed deaths from covid-19 to 11 April 2020, and weekly absolute number of deaths between 1 January and 4 April across recent years by age group and sex.

**Results:**

Nembro had 11 505 residents as of 1 January 2020. Monthly all cause mortality between January 2012 and February 2020 fluctuated around 10 per 1000 person years, with a maximum of 21.5 per 1000 person years. In March 2020, monthly all cause mortality reached a peak of 154.4 per 1000 person years. For the first 11 days in April, this rate decreased to 23.0 per 1000 person years. The observed increase in mortality was driven by the number of deaths among older people (≥65 years), especially men. From the outbreak onset until 11 April 2020, only 85 confirmed deaths from covid-19 in Nembro were recorded, corresponding to about half of the 166 deaths from all causes observed in that period.

**Conclusions:**

The study findings show how covid-19 can have a considerable impact on the health of a small community. Furthermore, the results suggest that the full implications of the covid-19 pandemic can only be completely understood if, in addition to confirmed deaths related to covid-19, consideration is also given to all cause mortality in a given region and time frame.

**Figure:**
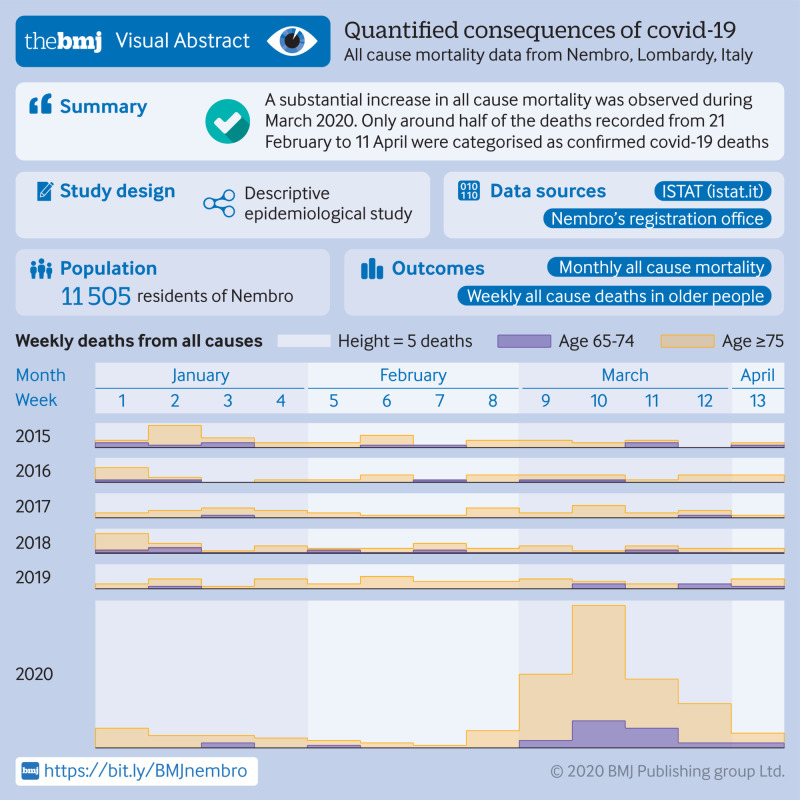


## Introduction

The global spread of severe acute respiratory syndrome coronavirus 2 (SARS-CoV-2) and the resulting coronavirus disease 2019 (covid-19)^1^ quickly escalated into a critical situation for healthcare systems worldwide and continues to pose a major threat to population health. In Italy, more than 28 700 people have died from covid-19, the highest number of officially reported covid-19 related deaths in Europe (as of 2 May 2020).^2^ ^3^ ^4^ The steep increase in the number of people with symptoms of covid-19 led to a sudden and catastrophic overload of Italian healthcare capacities.^5^ ^6^

The Lombardy region of northern Italy, an area with almost 17% of the Italian population (2019 data^7^), rapidly became the most severely affected area, and by late March the region comprised 60% of all covid-19 related deaths in Italy and 40% of the confirmed covid-19 cases.^8^ Considerable media coverage of Bergamo, one of the first Italian cities in Lombardy to be severely affected by covid-19, showed military vehicles carrying coffins to other cities because of the lack of space and morgue staff.^9^

Although the reported number of covid-19 related deaths in Bergamo is high,^10^ the real figure could be even higher according to all cause mortality data. A local investigation raised initial doubts about the accuracy of confirmed case and death counts, indicating a substantial underestimation of the magnitude of the burden.^11^ Such underreporting is not surprising given the state of emergency in many of the hospitals, the large number of patients needing immediate intensive care, the enormous time and emotional pressure on medical teams, the shortage of materials and human resources, and the clinical complexity of the disease.^5^ ^12^ ^13^ However, underreporting is not the only possible explanation for the considerable difference between the number of covid-19 specific deaths and the increase in all cause deaths.

The impact of covid-19 on all cause mortality is especially noticeable when data are analysed from small cities characterised by stable age-sex structures over time and low mobility. This metric is sensitive to small increases in absolute numbers of deaths in small cities. In an effort to accurately determine the consequences of covid-19 on mortality, we describe the change in all cause mortality over time in Nembro, a small city in the province of Bergamo (Lombardy) that has been severely affected by the covid-19 pandemic.

## Methods

### Setting

Nembro, located in the Bergamo province of Lombardy, has a population of 11 505 (2020 data^14^). In 2018, life expectancy at birth in the province was 81.2 years for men and 85.5 years for women, similar to those in the region.^7^ Between 2009 and 2015, cancer was the leading cause of death in men in the province (cause specific mortality 328.7 per 100 000 person years) and cardiovascular disease was the leading cause of death in women (317.8 per 100 000 person years).^15^ Among cancer related deaths, bronchial and lung cancers were the most common causes in men and the second most common causes in women.^15^ ^16^ Between 2009 and 2015, the mortality rate for all cancers in the province was higher than the rate in the region of Lombardy, while the mortality rates for cardiovascular diseases and bronchial and lung cancers did not differ.^15^ ^16^

In a provincial survey between 2011 and 2014 among adults aged 18 to 69 years, 24% were current smokers, 19% were former smokers, and 57% were non-smokers.^16^ Given the advanced age of the population, the province of Bergamo has a high prevalence of chronic conditions (especially hypertension, diabetes, and hypercholesterolaemia).^16^ When requiring medical care, most residents in the province of Bergamo were treated in hospitals close to home. The province ranked first among Italian provinces for having the lowest number of residents (1.85%) discharged from hospitals outside of the region.^17^

Indeed, the healthcare system in Lombardy is characterised by high standards and plentiful resources, with more than 200 accredited hospitals employing about 130 000 skilled healthcare workers.^18^ In this region, the capacity of intensive care units before the pandemic was about 720 beds (typically operating at 85-90% occupancy during winter).^6^ ^13^

By the end of February 2020, Nembro was one of the first Italian cities to report patients with covid-19 outside the original red zone around Lodi city. The first community isolation measures in Lombardy were implemented on 23 February, such as school closures, reduced commercial activity, and the cancellation of events and large gatherings. On 2 March, as a result of the emerging numbers of confirmed cases of covid-19, the Italian National Institute of Health recommended the creation of a red zone in the area, including Nembro.^19^ ^20^ These recommendations, however, were first implemented on 8 March; thereafter, no one could enter or leave the region and residents could not leave their homes, except for certain types of essential work or necessities such as groceries.^13^ ^21^ At the end of March, the mayor of Nembro publicly reported about 200 confirmed cases of covid-19 in the city.^20^

### Data sources

Our study data integrated information from multiple sources. Firstly, we retrieved publicly available information from the Italian National Institute of Statistics (ISTAT), a public organisation that provides official statistics for Italian citizens and policy makers. The ISTAT data we used are freely available.^7^ We extracted information on the number of Nembro residents at the beginning of each month from January 2012 to December 2019 and the number of residents who died from all causes each month from January 2012 to November 2019.

As a second source of information, we used data from Nembro’s official registration office. We obtained special authorisation from the mayor to receive anonymised information from this registry on the number of residents who died from all causes between 1 January 2015 and 11 April 2020. Because local authorities are rapidly informed about deaths, both in and out of hospitals, we believe this registry to be an accurate and direct information source for December 2019 and early 2020. Using a report issued from this office, we further extracted the number of residents in Nembro as of 1 January 2020.^14^

The number of confirmed covid-19 deaths in the city was obtained from a public repository^22^ provided by OnData,^23^ an association that promotes transparency and open data. OnData reported extracting this information from the official Lombardy region covid-19 map dashboard.^22^ From this source we obtained information from 21 February (the date of receipt of the first positive laboratory sample) until 11 April for those people living in Nembro who tested positive for covid-19. The dates from this source indicate when the biological sample was received by the laboratory, and the geographical reference listed is the city in which those people lived at the time they were tested (we did not anticipate this to differ meaningfully from the registered city of residence).^22^ From the OnData repository, we obtained information on the vital status of people living in the city who tested positive for covid-19; however, we were unable to discern whether those who tested positive actually died from the disease of interest or from another cause.

We also used data from the recently published ISTAT mortality dataset for covid-19 emergency, available for selected municipalities.^24^ From these data we extracted information about the weekly absolute number of all cause deaths by age group and sex in Nembro from 1 January to 4 April, for each year from 2015 to 2020.

### Statistical analysis

To estimate the amount of total person time Nembro residents spent at risk, we combined two approaches. Firstly, between 1 January 2012 and 1 January 2020, we interpolated values between the recorded population size at the beginning of each month, assuming the change between the two consecutive time points was constant within that interval. To do this we used a spline regression model with the population size on the first day of the month as the dependent variable and the time in days as the independent variable, which was transformed using linear splines with knots set to the first day of each month.

Since no data were available on the number of residents after 1 January 2020, for each day thereafter we estimated the number of residents using a weighted average of projections obtained from two models: the prolongation of the last segment of the spline regression and a third order polynomial linear regression fitted over the entire observed interval. To estimate the daily number of residents after 1 January 2020, we used a convex combination of the two projections with weight equal to the reciprocal of the square root of the elapsed days since 1 January 2020 to avoid unnatural jump discontinuity in the function and to account more for long term trends than for short term trends.

Using this strategy, we estimated the number of residents across the entire study period (1 January 2012 to 11 April 2020). We estimated the total person years spent at risk each month by summing the estimated number of residents each day of the month divided by 365.25. Our approach to compute person time between 1 January 2012 and 1 January 2020 is about equivalent to the established practise of estimating the person time for each month as the product between the month’s length and the average of the number of residents at the beginning and end of the month.

As a sensitivity analysis, we also estimated the monthly mortality rates under the hypothetical scenario of a large decrease in contributed person years during the final months of the study period (that is, to reflect many deaths or emigration, or both as a result of the pandemic). We projected the number of residents after 1 January 2020 in an alternative way by prolonging the last segment of the spline regression until 20 February 2020. From that day onwards, we estimated the number of residents by subtracting one tenth of the square of the elapsed number of days since 20 February from the estimated population size at 20 February.

We computed monthly all cause mortality rates by dividing the number of deaths by the estimated number of person years, expressed per 1000 person years. The April 2020 mortality rate was computed using data only from 1 to 11 April. We additionally compared the number of deaths captured by both the ISTAT covid-19 emergency dataset and the city registration office of Nembro from 1 January 2020 to 4 April 2020. Analyses were conducted using R version 3.6.0 and RStudio version 1.1.456.

### Patient and public involvement

No patients were directly involved in this study. After the study was conceived, additional data were obtained from the mayor of Nembro, and he is interested in the wider dissemination of these results.

## Results

**Fig 1:**
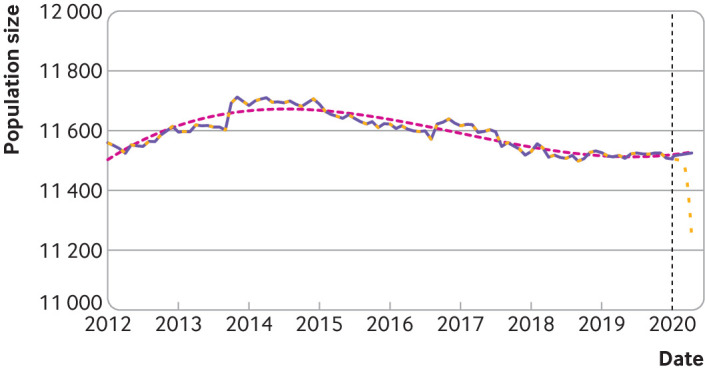
Population size of Nembro from 1 January 2012 to 11 April 2020. Actual numbers of residents were recorded for the first day of each month between 1 January 2012 and 1 January 2020. Beyond this date (black vertical dashed line), a convex combination of two projections was used to estimate the number of residents to 11 April 2020. The two projections were obtained from the last segment of the spline regression (purple line until 1 January 2020) and a third order polynomial linear regression fitted on the whole observed time period (dotted pink line). The purple line represents the population size used to estimate person years in the main analysis, while the yellow dashed line represents the population size under the possible scenario of large numbers of deaths or emigration after 20 February 2020 (used in the sensitivity analysis)

Between 1 January 2012 and 1 January 2020, the monthly number of residents in Nembro ranged from 11 498 to 11 712 (fig 1). Information on population size was available to 1 January 2020, at which time the number of residents was 11 505. Thereafter the number of residents projected using our approach reached 11 525 on 11 April 2020 (fig 1).

Between 1 January 2012 and 11 April 2020, a total of 1116 people in Nembro died of all causes. Of these deaths, 112 (10.0%) occurred in 2012, 112 (10.0%) in 2013, 95 (8.5%) in 2014, 119 (10.7%) in 2015, 126 (11.3%) in 2016, 109 (9.8%) in 2017, 128 (11.5%) in 2018, 121 (10.8%) in 2019, and 194 (17.4%) in the first months of 2020 (until 11 April). Of the 194 deaths in the first months of 2020, 151 occurred in March alone. Between 21 February and 11 April 2020, a total of 166 deaths were recorded among the residents.

Of the biological samples received by the regional laboratory between 21 February and 11 April, 218 people later tested positive for covid-19. Of these positive tests, 85 were documented to belong to people who had died (last updated 16 April). Overall, the data source contained 64 135 confirmed cases of covid-19 in the Lombardy region; only 2% did not have recorded information on location.

**Fig 2:**
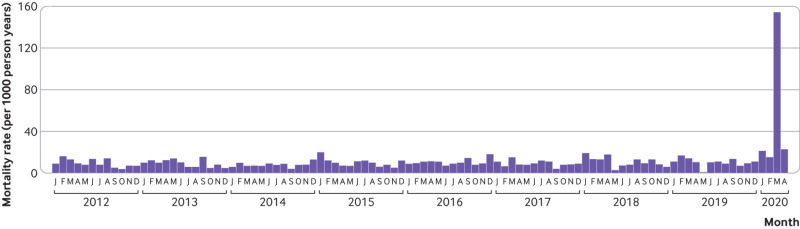
Monthly all cause mortality per 1000 person years in Nembro between January 2012 and April 2020 (data only available to 11 April). Initials represent the months

Monthly all cause mortality between January 2012 and February 2020 fluctuated around 10 per 1000 person years (range 1.0 to 21.5 per 1000 person years) (fig 2). In March 2020, monthly all cause mortality reached a peak of 154.4 per 1000 person years—the corresponding rate for the same month in 2019 was 14.3 per 1000 person years. In April 2020, based on data from the first 11 days, all cause mortality decreased to 23.0 per 1000 person years (fig 2).

Results from the sensitivity analysis accounting for a potential sudden decrease in the population size showed monthly all cause mortality rates of 15.3, 155.7, and 23.5 per 1000 person years in February, March, and April, respectively.

The number of deaths in 2020 began to rapidly increase during the week of 23 February, peaked during the week of 8 March, and subsequently declined until 4 April. Of the 161 people who died during this period, none were aged 14 years or younger and 14 (8.7%) were aged between 15 and 64 years. The deviation in weekly all cause deaths compared with previous years was largely driven by the increase in deaths among older people (≥65 years) and men (table 1). Among those aged 75 years and older, 47 deaths were observed during the week of 8 March alone, 33 of which were in men.

**Table 1:** Number of weekly all cause deaths among Nembro residents aged 65 and older between 1 January and 4 April during six years (2015-20) by sex and age group according to the ISTAT covid-19 emergency dataset

Week	65-74 years							≥75 years					
	2015	2016	2017	2018	2019	2020		2015	2016	2017	2018	2019	2020
**Women**													
01/01-11/01	0	0	0	0	0	0		1	1	1	3	1	6
12/01-18/01	1	0	0	1	0	0		5	1	2	2	3	4
19/01-25/01	1	0	0	0	0	1		2	0	3	0	1	2
26/01-01/02	0	0	0	0	0	0		1	0	2	3	2	2
02/02-08/02	0	0	0	1	0	0		0	0	2	0	0	1
09/02-15/02	1	0	0	0	0	0		3	2	1	1	4	2
16/02-22/02	0	1	0	1	0	0		0	0	0	3	1	1
23/02-29/02	0	0	0	0	0	0		2	1	3	1	3	2
01/03-07/03	0	1	0	0	0	2		2	1	1	3	3	16
08/03-14/03	0	1	0	0	1	2		2	1	4	1	1	14
15/03-21/03	0	0	0	1	0	0		1	0	1	0	1	10
22/03-28/03	0	0	1	0	1	0		0	2	2	2	0	12
29/03-04/04	0	0	0	0	1	0		0	2	1	1	2	3
**Men**													
01/01-11/01	2	1	0	1	0	0		0	4	1	4	1	2
12/01-18/01	0	1	0	1	1	0		3	0	1	0	0	1
19/01-25/01	1	0	1	0	0	1		0	0	0	1	0	1
26/01-01/02	0	0	0	0	0	0		1	1	1	0	2	2
02/02-08/02	0	0	0	0	0	1		2	1	0	1	2	1
09/02-15/02	0	0	0	0	0	0		1	1	0	1	1	0
16/02-22/02	1	0	0	0	0	0		0	0	1	0	2	0
23/02-29/02	0	0	0	0	0	0		1	2	1	1	0	5
01/03-07/03	0	0	0	0	0	1		1	1	1	0	1	11
08/03-14/03	0	0	0	0	1	9		0	1	1	0	0	33
15/03-21/03	2	0	0	0	0	8		0	1	1	2	1	10
22/03-28/03	0	0	0	0	1	2		0	1	0	0	0	4
29/03-04/04	1	0	0	0	0	2		1	1	0	1	1	1
**Total**	10	5	2	6	6	29		29	25	31	31	33	146

ISTAT=Italian National Institute of Statistics.^24^

No differences in weekly death counts were observed for 1 January to 4 April 2020 between the ISTAT covid-19 emergency dataset and the one used to compute mortality rates in our analysis.

## Discussion

This study found a steep increase in all cause mortality in Nembro in early 2020 compared with the rather stable mortality rate observed over the past eight years in this city. More Nembro residents died in March 2020 than in the entire previous year or in any single year since 2012, with the all cause mortality rate in that month almost 11 times that observed in March 2019. After accounting for a potential sudden decrease in population size in a sensitivity analysis, this deviation was even more pronounced. The increase in mortality was mostly driven by an increase in deaths of older people (≥65 years), especially men. Since the population of Nembro had been relatively stable across recent years, we conclude that this rapid increase in deaths is attributable to the covid-19 pandemic. Only about half of the deaths observed since the pandemic onset (21 February to 11 April 2020), however, were categorised as confirmed covid-19 deaths.

### Strengths and limitations of this study

The information used in our study was obtained from various sources. We acknowledge that some of the data might be provisional or not fully updated. Given the state of emergency and rapid development of covid-19, however, this limitation was unavoidable, and we did not observe any meaningful mismatches between the data sources. The source for confirmed covid-19 deaths is not official and does not include date of death, but rather the date the laboratory received the biological sample. This means the number of confirmed covid-19 deaths reported in our study is likely to be slightly higher than the official number in the same period because we included deaths of those who might have died after the 11 of April (although their sample was sent to the laboratory earlier). Furthermore, we know these deceased individuals tested positive for covid-19, but we cannot be absolutely certain whether the disease was a contributing cause of death. This means the difference between covid-19 specific deaths and the increase in all cause deaths could be even more extreme. The OnData repository we used in our study was the only data source available at the municipality level.

We did not estimate age-sex specific all cause mortality because information on the age-sex structure for Nembro was only available yearly, and the last update was on the 1 January 2019. Therefore, we preferred to avoid unreliable projections of person time at risk that would be based on strong, likely unreasonable assumptions.

### Comparison with other studies

Our findings corroborate results from a large study by the Istituto di studi e ricerche Carlo Cattaneo.^25^ In that investigation encompassing more than 1000 Italian cities selected because of an increase in mortality compared with previous years, the authors compared the overall number of deaths between 21 February 2020 and 21 March 2020 with the number of deaths in the same period averaged over the previous five years.^25^ The study concluded that even under the best case scenario, in which all other Italian municipalities (about 7000) showed no deviation from the average mortality measured in previous years, the number of deaths attributable to covid-19 in Italy would still be twice as high as the number of confirmed deaths from covid-19 reported by the Italian authorities.^25^ This study sheds light on the scale of the problem—that many deaths are erroneously not being attributed to covid-19 and that many of those who die outside of a hospital and have the disease are not being tested.^25^ The authors also noticed large increases in mortality in regions not considered to be key Italian SARS-CoV-2 hot spots.^25^ Another report, issued by the Italian Ministry of Health and the Italian National Center for Prevention and Control of Disease, about the daily surveillance mortality project (SiSMG) involving 18 major Italian cities, found similar results.^8^ According to this report, the two included Lombardy cities (Milan and Brescia) showed a large increase in the number of all cause deaths in the period from the beginning of the outbreak to 18 March 2020 compared with the average number of deaths in the same period across the previous five years.

### Conclusions and implications

Across Italian cities, all cause mortality has notably increased because of the covid-19 pandemic, but this increase is not being completely captured by officially reported statistics on confirmed covid-19 deaths. We believe several factors might have contributed to the discrepancy between the burden described by the confirmed death counts for covid-19 and that described by the increase in all cause mortality.

Firstly, covid-19 related deaths are generally counted as such if people test positive for the disease. Given the higher case fatality of covid-19 among older people with comorbidities, as well as the shortage of healthcare resources, many who actually died from covid-19 were likely never tested; therefore, the cause of death in these people was misclassified. For example, a shortage of tests prevented the assessment of covid-19 in people with symptoms and confirmed contacts in Nembro.^26^

A second explanation for the mismatch between these two death counts could lie in the group who did not have covid-19 but experienced other serious medical conditions and died from causes indirectly related to covid-19. During this period, this group might have experienced restricted access to healthcare owing to shortages in capacity, limited human resources for such a large patient influx (10% of patients with confirmed covid-19 in Italy worked in healthcare^27^), or fear of seeking hospital care during the pandemic. In Nembro, signs of the burden on the healthcare system and logistical challenges were noticeable.^26^ A recent article describes the challenges and difficulties of the provincial healthcare system to provide even basic healthcare services.^28^

Thirdly, the known delay between administering and processing the test and the availability of results, especially in overwhelmed settings, might have exacerbated this difference.

Our results describe the impact of the covid-19 pandemic on the health of a small community. On a larger scale, the consequence of an uncontrolled SARS-CoV-2 outbreak in Italy would be the collapse of the healthcare system,^13^ which, in turn, would have a substantial negative impact on the health of the entire population. We emphasise that measures of lethality are hardly interpretable solely as characteristics of the disease but also depend on the continuous availability and quality of care. The consequences of a pandemic are not only limited to covid-19 related deaths but rather contribute in an indirect way to the potentially avoidable deaths due to extreme triage of limited resources in crisis situations.^29^

Despite being weakened by a substantial reduction in public funding during the past decade,^6^ the Italian healthcare system’s overall performance still ranks high in international comparisons.^30^ However, in the face of the unprecedented challenge from covid-19, policy makers are only equipped with the ability to introduce social distancing measures to slow down the spread of the virus and protect vulnerable groups and simultaneously strengthen the healthcare system to ensure high quality care for all patients.^31^ ^32^ Our results showed a decrease in all cause mortality in early April 2020, possibly attributable to reduced spread of the virus and reduced case fatality. Potential explanations for reduced spread of the virus include the implemented stringent community isolation measures, the promotion of preventive behaviours, as well as a growing number of immune people. For the reduced case fatality rate, possible contributing factors include a smaller pool of vulnerable people as well as boosted healthcare capacities from reallocation and optimisation of resources.

Our findings imply that the reporting of confirmed covid-19 specific deaths represents, at least for some Italian regions, a substantial underestimation of the actual number of deaths from the disease. As a consequence, we believe data on all cause mortality should be considered along with traditionally reported measures as an important metric to evaluate and compare the consequences of the covid-19 pandemic within and between settings. Although all cause mortality can only be interpreted as an approximation of the health status of the population under study, it is more often systematically collected under high quality standards, relies on universally accepted classification, and is not influenced by testing strategies or shortages of tests. 25 Furthermore, this metric captures indirect deaths, such as those related to a healthcare system under crisis, yielding a more complete picture of the pandemic’s effects on population health. As we have outlined, this metric has several advantages and overcomes major drawbacks of other statistics for quantifying the impact of the covid-19 pandemic.

**What is already known on this topic**

The global spread of coronavirus disease 2019 (covid-19) has severely affected northern Italy
The consequences of covid-19 are generally assessed using the number of confirmed covid-19 related deaths

**What this study adds**

The covid-19 pandemic had a substantial impact on the health of the small community of Nembro city (Lombardy, Italy) based on comparisons of monthly all cause mortality since 2012
All cause mortality represents an important metric to quantify the burden of a pandemic

## Notes

Contributors: MP and TK conceived the study. LF and CL acquired and managed the data. MP analysed the data and created the figures. MP, JLR, and TK interpreted the results, and LF and CL provided important local insights. MP, JLR, and TK drafted the first version of the manuscript. TK supervised the work. MP is the guarantor and accepts full responsibility for the work and the conduct of the study, had access to the data, and controlled the decision to publish. All coauthors provided critical comments and approved the final version of the manuscript. The corresponding author attests that all listed authors meet authorship criteria and that no others meeting the criteria have been omitted.

Ethical approval: Not required. Most data sources used were fully publicly available. Data obtained with permission from the Nembro city registration office were fully anonymised.

Data sharing: Most of the data used in this study are freely available from the sources cited. The most recent data obtained from the Nembro city registration office is available upon reasonable request from Caterina Lurani at caterina.lurani@cmsantagostino.it. The analysis script is available from the corresponding author at marco.piccininni@charite.de.

Dissemination to participants and related patient and public communities: Upon request, the data will be available for policy makers and government bodies. We are sharing the results with the mayor of Nembro, for further dissemination within the city.

MP (the manuscript’s guarantor) affirms that the manuscript is an honest, accurate, and transparent account of the study being reported; that no important aspects of the study have been omitted; and that any discrepancies from the study as originally planned (and, if relevant, registered) have been explained.
